# Positive Feedback Loops in Alzheimer’s Disease: The Alzheimer’s Feedback Hypothesis

**DOI:** 10.3233/JAD-180583

**Published:** 2018-10-16

**Authors:** Andrew J. Doig

**Affiliations:** Division of Neuroscience and Experimental Psychology, School of Biological Sciences, Faculty of Biology Medicine and Health, Oxford Road, University of Manchester, UK

**Keywords:** Aggregation, amyloid, amyloid-β protein precursor, directed acyclic graph, drug discovery, peptide, systems biology

## Abstract

The dominant model for Alzheimer’s disease (AD) is the amyloid cascade hypothesis, in which the accumulation of excess amyloid-β (Aβ) leads to inflammation, excess glutamate and intracellular calcium, oxidative stress, tau hyperphosphorylation and tangle formation, neuronal loss, and ultimately dementia. In a cascade, AD proceeds in a unidirectional fashion, with events only affecting downstream processes. Compelling evidence now exists for the presence of positive feedback loops in AD, however, involving oxidative stress, inflammation, glutamate, calcium, and tau. The pathological state of AD is thus a system of positive feedback loops, leading to amplification of the initial perturbation, rather than a linear cascade. Drugs may therefore be effective by targeting numerous points within the loops, rather than concentrating on upstream processes. Anti-inflammatories and anti-oxidants may be especially valuable, since these processes are involved in many loops and hence would affect numerous processes in AD.

## THE AMYLOID CASCADE HYPOTHESIS

**Fig.1:**
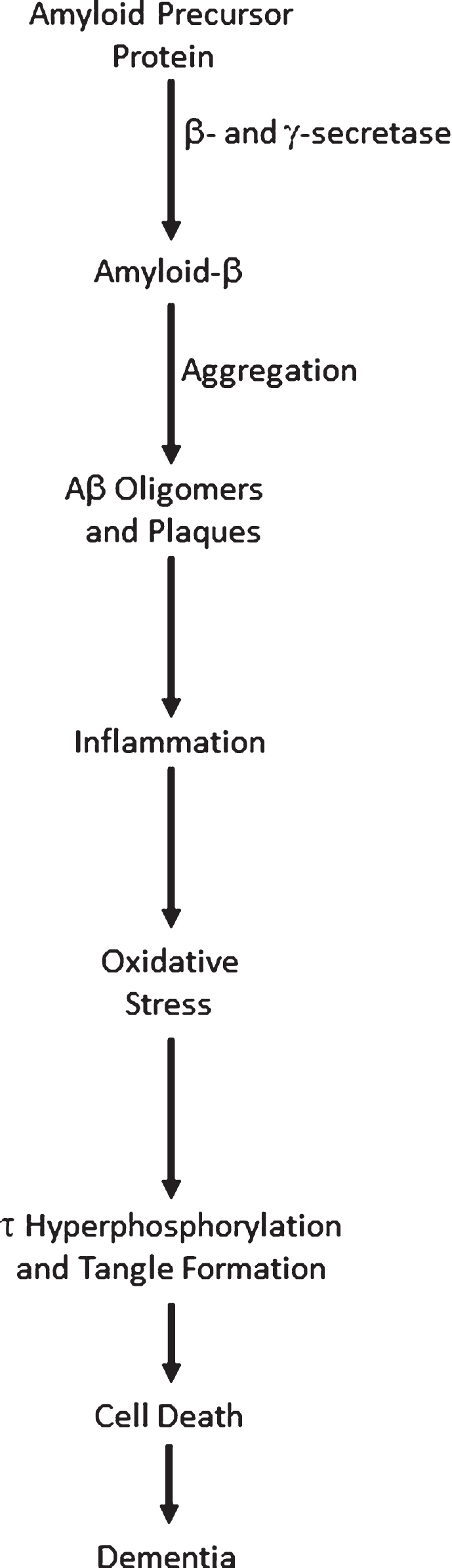
The Amyloid cascade.

Since 1992 [1], the amyloid cascade hypothesis has played the prominent role in seeking to explain the onset, progression, and pathogenesis of Alzheimer’s disease (AD). It proposes that the over-production and/or decreased degradation and subsequent deposition of amyloid-β (Aβ) is the initial pathological event in AD. A range of subsequent events then follow: Aβ oligomer and plaque formation, activation of microglia and astrocytes, chronic inflammation, increased levels of the neurotransmitter glutamate, elevated intracellular calcium, oxidative stress, synaptic dysfunction, tau hyperphosphorylation and tangle formation, neuronal loss, and ultimately dementia. A simple, though typical, representation of the amyloid cascade is shown in Fig. 1. A wealth of evidence supports the involvement of all these processes, and more, in AD. While there is thus little doubt that each of the steps shown in Fig. 1 are key events in AD, it is now apparent that numerous positive feedback loops also occur, requiring modification of the amyloid cascade hypothesis to a system of loops, rather than a linear process.

A cascade is defined as: “a small waterfall, typically one of several that fall in stages down a steep rocky slope”, or, more pertinently here, “A succession of devices or stages in a process, each of which triggers or initiates the next.” (https://en.oxforddictionaries.com/definition/cascade). Both these definitions imply that information flow can only travel in one direction, as water can only flow downhill, otherwise known as a directed acyclic graph. The amyloid cascade is thus a unidirectional process, where initial stress causes an increase in total Aβ (Aβ_42_ in particular), leading to a succession of processes and eventually dementia. In this model, perturbations to the healthy system can only lead to changes downstream of the location of the perturbation. For example, increased oxidative stress could cause tau hyperphosphorylation and tangle formation, but not remove Aβ oligomers. This concept of unidirectional flow of information is inaccurate, however, since it is now apparent that there are a variety of positive feedback loops in AD, where processes downstream in the cascade can stimulate processes upstream, leading to amplification of the initial perturbation. It is thus no longer tenable to frame AD as a cascade. Here I give examples of positive feedback loops in AD to illustrate this. The aim of this review is not to give a comprehensive overview of AD pathways— rather it is to show that diverse positive feedback loops are present in AD and to discuss their implications for the disease and its possible therapies.

## Aβ PEPTIDES AND AβPP FRAGMENTS

Aβ_40_ and Aβ_42_ are generally regarded as the most important amyloid-β protein precursor (AβPP) derivatives: Aβ_40_ because it is the most abundant in senile plaques and Aβ_42_ because it is also abundant and more toxic than Aβ_40_. Hence the Aβ_42_:Aβ_40_ ratio is often seen as a critical measure of AD stress, in addition to absolute concentrations of these peptides. Very many other AβPP fragments and derivatives are also likely to be involved in AD progression, however, such as soluble AβPP *α* and β (sAβPP*α* and sAβPPβ), C83, C99, P3, amyloid precursor protein intracellular domain (AICD), N3-pyroglutamate [2], isoaspartyl, phosphoserine and other post-translational modifications, and Aβ extended at both N- and C-termini [3, 4]. Even pure Aβ_42_ can aggregate into a host of oligomers (dimers, trimers, tetramers, hexamer, dodecamers etc.) with most likely multiple structures for each oligomer. We thus have a vast host of Aβ derivatives, with varying N- and C-termini, aggregated species of different sizes, and oligomers formed from more than one type of peptide, of differing populations, lifetimes, potential interactions with other molecules and toxicities. Hunter and Brayne have summarized how some of these can interact with each other in many ways, providing numerous feedback loops even when just considering interactions between Aβ species [5]. Aggregation is highly dependent on concentration, solution conditions, interactions with other molecules (e.g., seeds) and often shows variable lag times waiting for the formation of an aggregation nucleus. We thus have a highly complex situation, even considering only AβPP products *in vitro*. Hunter and Brayne discuss many of these issues in more detail [6]. How closely we can model the brain of a human with dementia, using a mouse model overexpressing an AβPP derivative, or cells with Aβ added extracellularly, remains to be seen.

## OXIDATIVE STRESS

Oxidative stress arises when there is an imbalance in the generation of reactive oxygen species (ROS), such as hydrogen peroxide (H_2_O_2_), hydroxyl radical (HO^·^), and superoxide (O_2_^·^^–^), over their removal. Life in an environment exposed to O_2_ causes a constant production of ROS. There is evidence for the complex involvement of oxidative stress at numerous points in AD pathogenesis [7–9], making it unclear to what extent oxidative stress is a cause or consequence of AD [9]. Metal ions such as zinc, iron, and copper have been found in amyloid plaques [10]. In particular, iron and copper can generate ROS, since they commonly exist in oxidation states differing by a single electron, thus readily generating free radicals. Elevated levels of Aβ have been reported to be associated with increased levels of oxidation products in proteins, lipids, and nucleic acids in AD hippocampus and cortex [11]. Such oxidation products may include Aβ itself, in the form of the sulphoxide at Met35 [12] or dityrosine linked dimers [13]. Mitochondria are major sources of ROS [14] and mitochondrial dysfunction may play a major, early role in AD [15–18]. Increased levels of Aβ may disrupt the electron transfer chain, increasing ROS production [19–21].

Some antioxidants, such as epigallocatechin-3-gallate (EGCG) and curcumin, have been reported to show beneficial effects in AD models and to decrease Aβ deposition [22–26]. These could work by multiple mechanisms, including: binding to Aβ directly, chelating metals [27], and acting as reducing agents or free radical scavengers. As such, disentangling the effects of anti-oxidants on AD pathways is difficult.

Here, I concentrate on evidence for feedback via oxidative stress in AD. There are several plausible mechanisms for this:

### LRP1

Low density lipoprotein receptor-related protein 1 (LRP1) is involved in receptor-mediated endocytosis. In particular, it helps clear Aβ from the brain to the blood, across the blood-brain barrier [28–30]. LRP activity is compromised by increased levels of oxidized LRP, which does not bind Aβ [31]. Levels of protein oxidation can be monitored by protein carbonyls and protein resident 3-nitrotyrosine (3NT); lipid peroxidation can be monitored by protein-bound 4-hydroxy-2-nonenal (HNE). LRP1 is oxidized in AD, shown by a 60%increase in the levels of the HNE-bound LRP1 β-subunit in AD hippocampus compared to age-matched controls, though there is no change in 3NT levels [32]. Aβ induced oxidative stress thus oxidizes LRP1, decreasing LRP1 activity, disrupting Aβ clearance [32] and causing Aβ accumulation in the brain.

### Oxysterols

End-products of cholesterol metabolism, named oxysterols, have been implicated in a wide range of diseases, including: atherosclerosis, inflammatory bowel diseases, age-related macular degeneration, and other pathological conditions characterized by altered cholesterol uptake and/or metabolism [33]. Non-enzymatic oxidative stress can form the oxysterols 7-ketocholesterol, 7*α*-hydroxycholesterol, 4β-hydroxycholesterol, 5*α*,6*α*-epoxycholesterol, and 5β,6β-epoxycholesterol. These have been detected in postmortem human AD brain and changes in their levels are associated with AD progression [34, 35]. Plasma levels of 24-hydroxycholesterol may be a marker of brain atrophy in AD patients [36].

Specific oxysterols have been suggested to be involved in AD in several ways: 3β-hydroxy-5-oxo-5,6-secocholestan-6-al can be converted into its aldol form and then react with an amine in Aβ to form a Schiff base (C = N double bond) [37]. This covalent modification of Aβ increases its amyloidogenicity [38], leading to the formation of neurotoxic spherical aggregates [39].

The oxysterol 24-hydroxycholesterol is produced in the brain by cholesterol 24-hydroxylase [40]. In contrast, 27-hydroxycholesterol is mostly synthesized elsewhere and enters the brain by crossing the blood-brain barrier [41]. An increased ratio of 27-hydroxycholesterol to 24-hydroxycholesterol has been observed in AD brains [42] where increased brain levels of 27-hydroxycholesterol may affect the production of Aβ [34]. In a human neuroblastoma cell line (SK-N-BE) differentiated into neuron-like cells, 1 μM of both oxysterols induced AβPP overexpression and increased β-secretase activity, leading to amyloidogenesis [43].

### Advanced glycation endproducts (AGEs)

AGEs are proteins or lipids that are non-enzymatically glycated by sugars (typically glucose), a process enhanced by oxidative stress. AGEs are commonly found in ageing brains, particularly those with AD. Increased AGE levels may explain many features of AD, such as crosslinking in Aβ and tau, glial activation and neuronal cell death. AGEs produce superoxide radicals directly by the transition metal-catalyzed oxidation of glycated proteins. AGE specific receptors (RAGE) bind Aβ and are upregulated in AD [44, 45]. The interaction of AGEs with cells increases oxidative stress by binding of AGEs to RAGEs on the cell surface and subsequent diffusion of ROS across the membrane or by interactions between a radical producing enzyme (e.g., NADPH-oxidase) and RAGE [45]. The combination of AGEs and RAGE can cause oxidative stress, secretion of cytokines, TNFα, heme oxygenase-1, and the activation of nuclear transcription factor *κ*B (NF*κ*B) [46–48], thus linking ROS to inflammation [49] and forming another positive feedback loop. Stimulation of inducible nitric oxide synthase by cytokines not only results in the synthesis of toxic nitric oxide, but also modifies Aβ via nitration, where nitrated Aβ suppresses long-term potentiation of synapses more effectively than Aβ alone [50, 51].

### TRPM2 and zinc

Transient receptor potential melastatin-related 2 (TRPM2) is a Ca^2+^-permeable channel primarily located on the cell surface, activated by ROS. In hippocampal neurons, exposure to Aβ_42_ generates ROS via protein kinase C and NADPH-dependent oxidases. ROS induces activation of mitogen-activated protein kinases and poly(ADP-ribose) polymerase-1, generation of ADP-ribose and activation of the TRPM2 channel. This causes lysosomal dysfunction and Zn^2+^ release to the cytosol. Zn^2+^ is highly neurotoxic [52], enhancing oxidative stress by impairing mitochondrial function and inducing mitochondrial generation of ROS, thus forming a positive feedback loop, driving loss of lysosomal and mitochondrial function and ultimately neuronal death [53].

## INFLAMMATION

There is strong evidence for the presence of feedback loops in AD via inflammation. Chronic (neuro)inflammation is prevalent in AD [51, 54–56]. AD brains show inflammation at and around the sites of plaques, tangles and afflicted neurons. The aggregation and deposition of Aβ plaques cause activation of microglia and astrocytes, accompanied by overexpression of complement system. Long-term use of non-steroidal anti-inflammatory drugs (NSAIDs) shows a reduction in the incidence of AD [57–60], perhaps via feedback to decrease Aβ deposition. Links from inflammation to Aβ are now apparent via several systems:

### Aβ in the periphery

Platelets are anucleate cells responsible for hemostasis and thrombosis. Circulating blood platelets are the second largest source of the AβPP protein after the brain, accounting for around 90%of circulating AβPP and are responsible for the majority of Aβ released into the plasma [61, 62]. Proteolytic cleavage of AβPP in platelets can occur at the cell surface or in intracellular secretory pathways. sAβPPα and Aβ peptides are stored in α-granules until platelets are activated, which leads to exocytosis via calcium-dependent degranulation [63]. Increased Aβ levels in the periphery may lead to increased Aβ in the brain via transport across the blood-brain barrier (the controversial peripheral sink hypothesis [31, 64–66]).

Aβ interacting with endothelial cells leads to a plethora of inflammatory factors being released. Resultant effects include: reduced anti-thrombotic activity, apoptosis, angiogenesis and accelerated tau aggregation. RAGE are also upregulated leading to over expression at the blood-brain barrier. This causes an increase in Aβ peptide deposition in the cerebral intracellular space. Contact between Aβ peptides and platelets causes further Aβ_42_ release by platelets, giving positive feedback.

COX-2 is one of two cyclooxygenases found in humans (the other being COX-1), responsible for the production of prostaglandins. In AD, Aβ binding to endothelial cells increases the production and release of COX-2, with a subsequent increase in COX-2 concentration. An accumulation of COX-2 above normal levels is also seen in the regions of the brain associated with AD (cerebral cortex and hippocampus). This additional COX-2 is stimulated by inflammatory cytokines, mitogenic stimuli and glutamate, in addition to Aβ [67]. COX2-expressing cells show elevated levels of AβPP mRNA leading to increased Aβ synthesis [68]. The product of COX2 activity, the unstable PGH2, rearranges into PGE2 and PGD2, as well as into the highly reactive γ-ketoaldehydes levuglandin E2 and LGD2 [69]. These levuglandins prevent Aβ proteolysis and accelerate Aβ oligomerization [70].

In human platelets, thrombin and PGE2 regulate the processing and secretion of Aβ peptides and sAβPP. The thrombin receptor is activated by Aβ, which leads to a cascade of events resulting in the stimulation of cPLA2. cPLA2 catalyzes the release of arachidonic acid, allowing synthesis of TxA2, activation of platelets and release of Aβ_42_ [71, 72].

Aβ contact can also lead to membrane disorganization, shrinking of platelets, and induction of apoptosis via ROS and inflammatory responses. Continued platelet activation and Aβ release results in chronic inflammation and further endothelial cell stress [71]. Hence, platelets are likely to accelerate and increase the severity of AD [73], via several positive feedback loops.

### Inflammasome

Inflammasomes are innate immune system receptors and sensors that regulate the activation of caspase-1 and release of pro-inflammatory proteins IL-1β and IL-18, thus inducing inflammation in response to infection or disease modified molecules derived from host proteins [74]. In particular, inflammasomes can respond to Aβ [75, 76]. Human brains with AD or mild cognitive impairment show strongly enhanced active caspase-1 expression via NLRP3 inflammasome activity [77]. *Nlrp3*^–/–^ or *Casp1*^–/–^ mice carrying mutations associated with familial AD showed less memory loss, reduced brain caspase-1 and IL-1β activation, and enhanced Aβ clearance. NLRP3 inflammasome deficiency resulted in the decreased deposition of Aβ in the AβPP/PS1 model of AD [77], thus showing positive feedback between Aβ deposition and inflammation, via the inflammasome.

Intracellular inflammasome formation is followed by the release from microglia of complexes of the ASC protein called ASC specks. Recent work provides clear evidence for feedback from the inflammasome to Aβ deposition via ASC. ASC interacts directly with Aβ, as it readily seeds formation of amyloid-like aggregates *in vitro*, a process inhibited by ASC antibodies. AD mice genetically engineered to lack ASC had less deposition of Aβ in the brain and improved memory, compared to when ASC was present. Brain extracts from aged *A*β*PP*/*PS1* mice induced the formation of Aβ deposits in the brains of young *A*β*PP*/*PS1* mice, but only if ASC was present [78, 79].

### RIPK1

A positive feedback loop between inflammation and Aβ may also act via RIPK1 kinase. Aβ accumulation in mice activates RIPK1 in disease-associated microglia, causing lysosomal defects and impaired phagocytic clearance of extracellular Aβ by microglia, thus giving a positive-feedback loop between neurons and microglia. Activated RIPK1 also causes excessive release of TNFα and IL-6 cytokines [80, 81].

## GLUTAMATE

**Fig.2:**
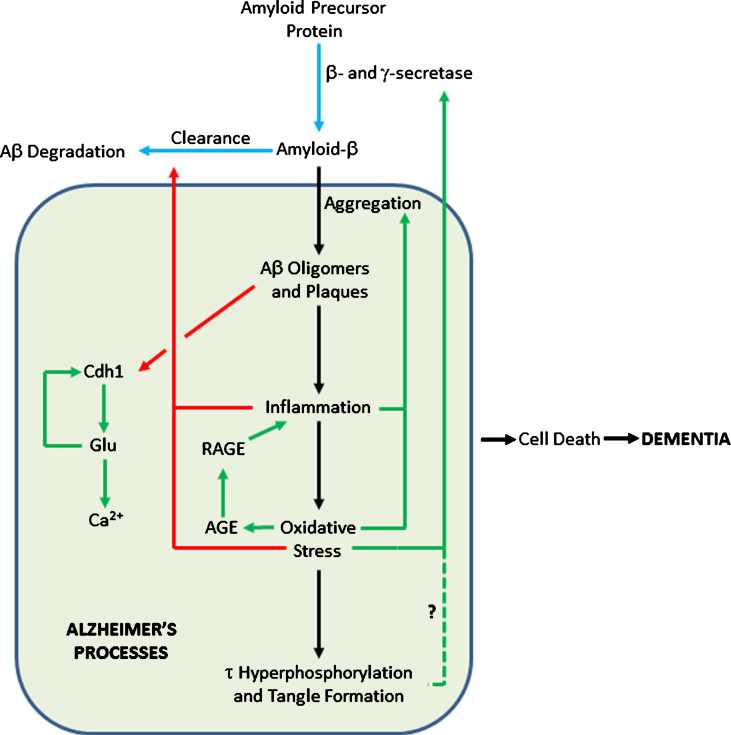
Feedback Loops in the Amyloid Cascade. Blue arrows show pathways in healthy cells; black arrows show AD pathways; pathways activated in AD are in green; pathways inhibited in AD are in red. The dashed line shows a tentative connection from hyperphosphorylated *τ* to increased AβPP processing to Aβ.

The anaphase promoting complex/cyclosome (APC/C) is a large protein complex forming an E3 ubiquitin ligase, targeting numerous proteins for degradation, particularly those involved in cell cycle regulation in proliferating cells [82, 83]. Cdh1 is an activator subunit of APC/C, expressed in post-mitotic neurons [84]. Aberrant accumulation of some of the targets of APC/C-Cdh1 has been associated with neurodegeneration [85]. For example, an increase in the APC/C-Cdh1 target cyclin B1 [86] affects control of the cell cycle in neurons [87]. Aβ oligomers cause proteasome-dependent degradation of Cdh1 in primary neuronal culture. This leads to a subsequent accumulation of glutaminase, an enzyme that converts glutamine to glutamate [88] and a degradation target of APC/C-Cdh1. Increased levels of glutaminase have been found in the prefrontal cortex of AD patients [89]. Elevated glutamate levels cause increases in intraneuronal Ca^2+^ levels, resulting in neuronal apoptosis. Excitotoxic glutamate stimulus causes APC/C-Cdh1 inactivation by decreasing Cdh1 [86]. This causes further accumulation of glutaminase, creating a positive feedback loop [88] (Fig. 2).

## CALCIUM

Ca^2+^ signaling plays many roles in cellular function as a second messenger, involving entry and release channels, clearance mechanisms, and intracellular stores. Ca^2+^ dysregulation plays a central role in AD [90–94].

Several positive feedback loops are present in AD that go through Ca^2+^, where increased Ca^2+^ promotes a pathogenic state, which in turn further increases Ca^2+^ levels [93]. Application of Aβ to cultured cells triggers flux of Ca^2+^ through the plasma membrane, either by acting on ion channels, disrupting membranes or forming pores [93, 95, 96]. Increased Ca^2+^ levels can lead to mitochondrial Ca^2+^ overload, oxidative stress by generation of superoxide radicals, and production of pro-apoptotic mitochondrial proteins such as caspases and cytochrome c [90]. As above, oxidative stress can promote the generation of more Aβ. In addition, mutations in proteins that control cytosolic Ca^2+^ concentrations are known to affect Aβ levels and susceptibility to AD [97].

## TAU HYPERPHOSPHORYLATION

The hyperphosphorylation of tau, leading to its dissociation from microtubules and formation of tangles, is invariably shown downstream of Aβ deposition. Much evidence from *in vitro* and *in vivo* models, and from biomarkers in patients, show Aβ-induced acceleration of tau pathology as a critical trigger for AD, though the exact mechanisms linking Aβ to tau hyperphosphorylation and accumulation are unclear [98–100]. AD is thus a disease of both Aβ and tau aggregation, whereas tauopathies, caused by mutations in the MAPT gene, show only tau pathology. Nevertheless, intriguing work on the effects of knocking out the MAPT gene in transgenic mice expressing mutant AβPP and PS1 suggest that tau may also be able to exert upstream effects on Aβ [101]. As would be expected if tau plays a key role in AD, AβPP/PS1/tau^++^ mice had reduced survival, neuronal and synaptic loss, and developed spatial memory impairments. These deficits were rescued in AβPP/PS1/tau^–/^^–^ mice, showing that tau contributes to AD pathology. Remarkably, however, levels of soluble and insoluble Aβ_40_ and Aβ_42_, the Aβ_42_/Aβ_40_ ratio and the amyloid plaque burden were decreased in the cortex and the spinal cord of the AβPP/PS1/tau^–/^^–^mice. Levels of phosphorylated AβPP, of β-C-terminal fragments (CTFs), and of β-secretase 1 (BACE1) were also lowered, suggesting that β-secretase cleavage of AβPP was reduced in AβPP/PS1/tau^–/^^–^ mice. The tau deletion therefore had a protective effect against amyloid induced toxicity, not just by reducing tau pathology directly, but also by reducing the production of Aβ, thus showing feedback from tau to Aβ production. Positive feedback may, therefore, reach all the way from tau to AβPP processing, provided that these results generalize from this mouse model.

## CONCLUSIONS: IMPLICATIONS FOR AD AND AD THERAPIES

The purpose of this review is not to give a comprehensive overview of all the pathways involved in AD. Rather, it is to show that there is compelling evidence for positive feedback loops in AD pathogenesis and hence that the simple concept of a cascade, with information flow only in one direction, is unsustainable.

Figure 2 shows where such loops can be added to the amyloid cascade. In particular, inflammation and oxidative stress can affect Aβ production, deposition, and clearance in multiple ways. Such loops may help explain the onset of the disease. Most people never get AD; the physiological processes relevant to AD are maintained in a state of homeostasis. For AD pathogenesis to start, it is likely that multiple stresses are needed to move the system far enough away from the healthy state so that positive feedback loops operate [102], committing the body to the AD pathway and subsequent dementia. The feedback loops mean that the body is in an either/or state of health or AD, since the cusp between the two states is unstable— either homeostasis or AD feedback loops will pull the system to one state or the other.

Multiple stresses, such as an ApoE4 genotype [103], age, inefficient protein degradation [104], autophagic/lysosomal dysfunction [105], iron dysregulation [27], deficiencies in blood coagulation via bacterial lipopolysaccharides [106, 107], vascular conditions [108], mitochondrial dysfunction [17], viral infection [109–111], microbial infection [112], and head injury [113], are almost certainly required to initiate progress into AD. Even dominant mutations that cause early onset AD are insufficient in themselves, as AD is not present from birth and decades of ageing are also necessary. Regarding AβPP as the single entry point to AD is thus an oversimplification, since factors such as prolonged mitochondrial dysfunction, impaired protein degradation or chronic inflammation can also be involved.

The presence of feedback loops shows that the term cascade is misleading, since it implies a unidirectional progression from upstream processes, such as overproduction and deposition of Aβ, to dementia. While Aβ undoubtedly plays a key role in AD, it is an oversimplification to think that Aβ causes all other processes in AD. Instead, a complex network of positive feedback loops reinforces AD processes once the body is committed to the disease. Given this complexity, a systems biology approach that quantifies fluxes within these pathways may well be essential to understand effects of perturbing the system [114–116]. Poorly liganded iron can generate hydroxyl radicals via positive feedback loops in many pathological conditions, including AD [117].

Multiple phenotypes associated with AD are deleterious. Hence, Fig. 2 shows that the totality of AD processes contributes to cell death, rather than there being a direct link to cell death only from tau, as in Fig. 1. Ideally, one would want to reverse all these phenotypes, restoring then to normal levels. Combinatorial therapies may be essential [117–119], since hitting a single target in a pathway may well have a negligible effect on its total flux [120].

If AD is a cascade, however, then interventions cannot affect upstream processes, if the steps are irreversible. Anti-inflammatories or inhibitors of the kinases that hyperphosphorylate tau would then not affect Aβ production and deposition, for example. A naïve interpretation of Fig. 1 is that drugs should seek to intervene as early as possible in the cascade to beneficially affect as many adverse phenotypes as possible. Intense work on β-and *γ*-secretase inhibitors, and antibodies targeting Aβ has implicitly followed this strategy, with little success to date.

Alternatively, if AD is driven by feedback loops, then it may not be the case that drugs should preferentially hit upstream targets. Many processes thought to be downstream of Aβ actually appear to affect AβPP processing, and Aβ deposition or clearance. Intervention at any point, or better still multiple points, in a loop may thus cause favorable phenotypic changes throughout the cascade. Blocking feedback loops may allow the body to escape AD processes and restore healthy homeostasis, by clearing excess Aβ once its overproduction is no longer stimulated. We should therefore perhaps take seriously a much wider range of drug targets in AD, particularly combinations of anti-inflammatories and anti-oxidants. Even targeting tau may be able to restore excess Aβ. This prospect of a much wider range of AD targets gives hope for developing new AD therapies.
